# “I’m Never Going to Be in Phantom of the Opera”: Relational and Emotional Wellbeing of Parkinson’s Carers and Their Partners in and Beyond Dancing

**DOI:** 10.3389/fpsyg.2021.636135

**Published:** 2021-07-29

**Authors:** Moa Sundström, Corinne Jola

**Affiliations:** ^1^Health Psychology Section, Psychology Department, IoPPN, King’s College London, London, United Kingdom; ^2^Division of Psychology, School of Applied Sciences, Abertay University, Dundee, United Kingdom

**Keywords:** quality of life, care partnerships, transferrable skills, motor representation, relationship, social interaction, action observation, dance rehabilitation

## Abstract

The caregiving of people who suffer from Parkinson’s predominantly falls on their life partners. Living with and caring for somebody with Parkinson’s can cause a range of emotional, psychological, and financial pressures. Whilst an increasing number of alternative treatments for Parkinson’s is available, such as dancing, the focus is predominantly on the motor and emotional improvements of the person suffering from Parkinson’s. For caregivers, however, dancing can be a double-edged sword: Although dancing can offer an opportunity to enjoy a social event with their partner; attending dance classes puts additional responsibilities on the carer. The present study thus aimed at exploring the experiences of participants with Parkinson’s who attended dance classes as well as the experiences of their care-partners in and around these classes along with their view on everyday life changes experienced since dancing. Six couples were interviewed individually where one partner had Parkinson’s. The interviews were also analyzed separately using inductive thematic analysis. In line with existing programmes that offer dance for people with Parkinson’s, the classes used a mixture of ballroom, ballet, contemporary, and creative dance styles; supported and influenced by an instructors’ extensive knowledge of the abilities and needs of those with Parkinson’s. A recurring challenge for Parkinson’s sufferers relates to “who is in control?” based on the many unknown changes of Parkinson’s; as well as seeing/being seen. Yet frustrations were oftentimes counteracted with humour. Also, when dancing, participants with Parkinson’s reported enjoying playful interactions. Caregivers’ themes focussed on theirs and their partners’ wellbeing regarding social contacts and openness, as well as issues surrounding their responsibilities as carers. Whilst some identified dance movements that help them in everyday tasks, they and their care-partners question the impact of dance on their motor control. Yet, participants unanimously agree that dance provides relevant opportunities for social contact and comparison. Nevertheless, the care-partners’ concerns remain about the burden of increasing responsibility for the wellbeing of both partners but they also reported enjoying dancing with their partner. Experiencing their loved ones as more cheerful after starting dance classes is recognised an important positive and impactful outcome of dancing together.

## Introduction

Parkinson’s disease is a progressive dopaminergic neurodegenerative disorder typically associated with physical symptoms such as tremors, freezing of gait, and a general functional slowness (Maffoni et al., 2017) leading to a greater risk of falls (Heiberger et al., 2011). Although these are common symptoms, there is a range of other manifestations such as sleep disturbances (Menza et al., 2010), facial masking (Wootton et al., 2018), forgetfulness (Khoo et al., 2013), and speech impairment (Ho et al., 1998). The physical symptoms are understood to have a dopaminergic cause and the medications available thus specifically target dopamine levels and receptors in the brain. However, this often does little to nothing for many non-motor symptoms, which usually are not directly linked to dopamine (Khoo et al., 2013). Further, research has shown that non-motor symptoms can have a significant detrimental effect on the person with Parkinson’s and those around them (see in Khoo et al., 2013; Soileau et al., 2014). For example, impaired cognitive functions, such as memory, and communication problems can make everyday interactions for Parkinson’s sufferers and their partners, family, and friends a challenge (e.g., Saldert and Bauer, 2017). This is particularly pertinent as partners or family members often also become an informal or formal carer for the person suffering from Parkinson’s.

Living with and caring for people with Parkinson’s can lead to caregiver burden, particularly as the disease advances and the person becomes more disabled, thus ever demanding more tasks from the carer (Drutyte et al., 2014). Caregiver burden can be defined as the negative physical and psychological effects a person experiences as a result of taking care of someone who is sick, usually a loved one (Yang et al., 2019). The is seldom only physical, but also financial (Santos-García and de la Fuente-Fernández, 2013), emotional, and social (Smith and Shaw, 2017). Research has also shown that the non-motor symptoms can have a greater negative impact on the care-partner and the relationship than the motor symptoms do (Hiseman and Fackrell, 2017). Notably, caregiver burden can have a harmful effect on both parts, to the extent that intervention is needed before the carer shows signs of burn-out. Their own health can influence their ability to care for their partner as well as themselves (Drutyte et al., 2014).

The field of research in care burden tends to paint a depressing picture of life post-diagnosis. Evidence suggests that carers’ higher stress-related morbidity is linked with their care duties (Glozman, 2004). It is indeed common for care-partners to suffer from sleep disturbances (Happe and Berger, 2002), feel depressed, anxious, fatigued, and stressed (Drutyte et al., 2014). Although life with Parkinson’s poses immense physical and emotional difficulties, accepting the diagnosis and this new way of life whilst making the most out of each day was found to help many couples live happy and fulfilled lives together (Smith and Shaw, 2017). What is, however, particularly challenging in relationships with people who suffer from Parkinson’s is that some of the Parkinson’s non-motor symptoms directly affect conditions that are basic for a successful interpersonal interaction. For a healthy relationship, partners need to be able not only to verbally communicate well, but to recognise and express emotions as well as feel a certain level of empathy toward one another (Fitness, 2015). Yet, people with Parkinson’s often show hypomimia, also described as facial masking, resulting through a loss of control of facial muscles and spontaneous facial expressions (Wootton et al., 2018). Another symptom of Parkinson’s is alexithymia, which is difficulty in recognising and verbalising one’s emotions (Ricciardi et al., 2015). Hypomimia and alexithymia interrupt the chain of emotional signalling in social interactions, which is confusing and frustrating (e.g., imagine being confronted with an emotionless expression in an emotionally social context). Indeed, facial masking has been found to increase the care burden, as the care-partner not only supports their loved one living with a deteriorating disorder but might also struggle to even recognise their partner of many years (Gunnery et al., 2016). Care-partners sometimes report feeling as if they are caring for a stranger, especially if the person with Parkinson’s experiences behavioral changes as well as difficulties with showing appropriate emotional responses (Drutyte et al., 2014; Mercer and Best, 2016). Further, a loss of empathy in Parkinson’s is generally a poorly recognised symptom, yet it significantly affects caregivers’ burden and mental health. Pomponi et al. (2016) showed that an improvement in Parkinson’s empathy levels through a 6-month intervention had a positive effect on their spouses’ wellbeing. As people with Parkinson’s struggle with their emotional regulation and are unable to express themselves romantically or positively, it is no surprise that care-partners feel depressed, lonely, and isolated. Indeed, the care-partner can be easily overwhelmed if their partner has depression and is also socially isolating themselves from other social support networks, further increasing the care burden as well as the risk of developing depression, a reduced overall quality of life, and early onset mortality (Drutyte et al., 2014; Mercer and Best, 2016). It is thus crucial to gain a better understanding of the impact non-medical interventions can have on people suffering from Parkinson’s as well as their caregivers. Based on the issues observed in regards to social isolation, depression, and social-interaction between life partners, a reduction in isolation and an increase in experience of emotional togetherness seems relevant for the wellbeing of caregivers in their role as carers and partners in response to an effective alternative treatment.

Over the past decade, there has been a surge in developing alternative treatments for Parkinson’s. Different forms of exercise and mindfulness programmes, such as Nordic walking (Cugusi et al., 2015), yoga (Ni et al., 2016), tai-chi (Li et al., 2012; Ghaffari and Kluger, 2014), or various forms of dancing (McNeely et al., 2015) found positive effects on gait, balance, coordination, slowing reduction of muscle mass, but also reduced levels of anxiety and depression. However, many complementary treatments are not covered by health insurances which enhances the couples’ financial burden. Moreover, caregivers or their Parkinson’s partners might feel anxious about their physical abilities. It is thus important to explore the lived experiences of care-partners of such body-mind programmes.

In this study, we focus on dance for Parkinson’s for three reasons. Firstly, dance classes are very popular amongst people with Parkinson’s. It is thus relevant to capture the experiences with dance classes of people with Parkinson’s as well as their caregivers. Secondly, whilst studies on dance for Parkinson’s consistently show positive effects on Parkinson’s emotional, embodied, and social wellbeing (e.g., Houston and McGill, 2013; Koch et al., 2016; Holmes and Hackney, 2017; Hadley et al., 2020), statistical evidence for motor advantages of dance compared to other interventions is mixed (see for example Rawson et al., 2019). Moreover, levels of frustration are frequently reported. For example, Bognar et al. (2017) participants revealed that dancing allowed them to create meaningful social connections both verbally and non-verbally through movement and music leading to an overall happier mood even though frustration at the inability to do certain movements occurred fairly regularly. Holmes and Hackney (2017) found that an adapted tango programme for people with Parkinson’s can improve their ability to carry out daily activities, improve their quality of life, create strong social bonds, but frustrations related to motor impairments were prevalent. Therefore, considering the inconsistencies in the reported motor changes in addition to the frustrations felt by the people with Parkinson’s, it is important to look at the care-partners’ lived experiences and their motivations to support their partner’s dancing. Thirdly, dance is a multifaceted activity for which various forms exist that were found to impact on emotional, psychological, and social aspects in addition to physical training (Jola and Calmeiro, 2017). Yet large heterogeneity across studies is challenging. Whilst the impact of dancing on people with Parkinson’s has been researched extensively through several forms of dancing, such as tango (Duncan and Earhart, 2014; Hackney et al., 2007), ballroom (Kunkel et al., 2017), ballet (Houston and McGill, 2013), or contemporary dance (Heiberger et al., 2011), we know little about what facets of dance lead to which outcomes (Jola, 2020). For example, Rocha et al. (2018) found in their randomised controlled trial some motor improvements in the Tango group and a reduction of freezing of gait in the mixed dance group. As a possible contributing factor, the authors emphasised the importance of the interaction of movement with music which is in line with many other studies (see Bek et al., 2020). Rocha et al. (2017, 2018) also highlight the importance of the instructors’ expertise in adapting the movements to the participants’ abilities in their studies; yet no other specific association between dance style and motor or non-motor improvement was proposed. It is also possible, for example, that the dancing is effective through the training of kinaesthetic sensation, emotional expression of the body and the face, or the opportunities to observe others, all of which are understood to enhance empathic interaction, as observed and discussed extensively in the dance movement therapy literature (e.g., Federman, 2011; Behrends et al., 2012; Shuper Engelhard, 2019). These aspects of dance training might thus be particularly effective for the wellbeing of couples where one suffers from Parkinson’s since verbal and non-verbal communication are severely affected by the disease. It is thus of great interest to identify which aspects of the dance classes are of importance for the dancing parties in and beyond these classes.

To our knowledge, no peer-reviewed publication investigated the specific effects a Parkinson’s dance intervention has on their caregiver spouses. Whilst Holmes and Hackney (2017) included care-partners as dance partners in their study, their experiences were not part of the study. Caregivers’ experiences were however part of the qualitative study by Rocha et al. (2017). The authors held a stakeholder forum to better understand the benefits and limitations of therapeutic dancing classes for people with Parkinson’s disease. Stakeholders included health clinicians, dance instructors, people with Parkinson’s and caregivers. The aim was to use the findings through thematic analysis in designing and implementing effective dance classes for people with Parkinson’s. The authors found that creative expression and a distraction from the disease through an immersion in dance as an art form were important features of the success of Parkinson’s dance classes. We understand that creative expression and a distraction from the disease are factors that might not only support people with Parkinson’s through dance participation, but also their spouses.

The aim of the current study was thus to explore the experiences of participating in a dance for Parkinson’s programme in people with Parkinson’s and their care-partners. What are the experiences in and around the dance classes that make them an increasingly common alternative treatment? What changes are noticed by carers and people with Parkinson’s in their everyday life together since they started dance classes? Which of these could be attributed to the dancing? In order to answer our research questions, a novel approach was adopted in that the couples were interviewed individually to make them feel more comfortable with sharing their personal experiences in and beyond the dance classes. Personal experiences reported separately may lead to a different emphasis than when discussing them in front of their partner.

## Materials and Methods

Ethical approval for the current study was received from the School of Applied Sciences Research Ethics Committee, Abertay University (EMS1835).

### Participants

Six couples were recruited using convenience sampling from two dance programmes for people with Parkinson’s in Greater London. The participants with Parkinson’s (five male) were aged 50–77 (*M* = 69.50, SD = 9.60) and their partners (five female) were aged 56–83 (*M* = 68.83, SD = 8.33). They were all in heterosexual relationships and had been married between 29 and 53 years (*M* = 41.83 years, SD = 8.84). The people with Parkinson’s had been diagnosed 5–25 years ago (*M* = 13.3 years, SD = 6.90 years). The inclusion criteria to participate in the research were: (1) to have had a Parkinson’s diagnosis for at least 1 year; (2) have no other neurological condition (e.g., dementia); (3) have participated in a minimum of 10 dance classes; and (4) have a partner who acts as a caregiver and is also willing to be interviewed. For the person with Parkinson’s, demographic information such as age, gender, time and stage of diagnosis, medication, and information on other medical conditions were recorded. All participants were taking one or more medications for Parkinson’s at the time of interviewing, and they were between stages 1–4 (*M* = 3.16, SD = 1.07) on the five-step Hoehn and Yahr scale (McRae et al., 2002) as assessed by the first author at that time point. All participants participated in the dance classes at least once a week and the participants with Parkinson’s were interviewed before the partners. All personal information was anonymous or pseudo-anonymised during the transcription phase using the letters from A to L for our 12 participants in alphabetical order (i.e., adjacent letters are couples). Since four participants with Parkinson’s were male, some information could have been easily identifiable. We thus used gender neutral pronouns in the text and replaced gender specific pronouns in the referenced statements with “they” or “them.”

### Dance Classes

Our participants were recruited from two dance programmes in the Greater London area, the South London Inclusive Dance Experience (SLiDE) and Move into Wellbeing. Whilst some teachers have trained in a Dance for PD workshop, these classes were not certified Dance for PD classes at the time of the study. The 1-h classes used a mixture of ballroom, ballet, contemporary dance styles with influences from tango and creative dance and the instructors’ extensive knowledge in teaching dance for people with Parkinson’s. The movements were continuously adjusted to the participants needs and abilities. Likewise, the music played by the instructors varied widely, from classical, modern pop, and rock and pop music from the 60s and 70s, mostly played through loudspeakers. At the time of the interviews neither of the classes had live music, although in the past one of the classes had had a pianist.

### Materials and Apparatus

A Homder digital voice recorder was used to record the interviews, and a pen and notepad were used during the interview to note down body language, facial expressions, mood, and other non-verbal factors and added to the transcripts in brackets as were clarifications on what participants talked about.

Two separate semi-structured interview schedules were used for the persons with Parkinson’s and their partners. The questions were aimed to evoke rich responses through talking about specific experiences but also included general questions to open-up the discussions. The decision to ask open questions in a non-suggestive manner was made to let the participants lead the conversation as the aim of the interview was to allow for a participant-led thematic framework to emerge on participants’ experiences surrounding the impact of dance on PD and their relationship. In other words, we were interested in how much the participant would bring up dance unprompted, and thus did not instantly ask about dance or ask too many direct questions about their dance experiences. Questions and answers were thus overall, relatively broad and covered a range of topic areas, from their experience with the disease, the dancing, the relationship, as well as questions related to their confidence or self-esteem (See Table 1). The most specific dance related questions the persons with Parkinson’s were asked were for example “Can you remember a moment when you really enjoyed the class? What was happening? How did it make you feel?” but we also asked “What does a Parkinson’s diagnosis mean to you?” to understand how the experience of Parkinson’s may be associated with the view on dance or the relationship with their partner. The care-partners were also asked semi-structured interview questions such as “Can you remember a moment that gave you a positive feeling since your partner started dance classes? Imagine being in that moment, what was happening? How did it make you feel?” To ask participants to remember specific moments and to talk freely about a situation and related contexts is an approach that is common in qualitative research practices (Vaismoradi et al., 2013).

**TABLE 1:** Sample interview questions and prompts.

**Experience**	**Questions for people with Parkinson’s**	**Questions for care-partners**
Parkinson’s	What does a Parkinson’s diagnosis mean to you? In what ways does Parkinson’s affect your everyday life? Have you had a difficult experience in public? Experienced stigma? How has Parkinson’s changed your social life? What are some of the symptoms you’re dealing with? What did you know about Parkinson’s before you got diagnosed?	What does a Parkinson’s diagnosis mean to you? What are some of your partners symptoms you noticed, before and after the diagnosis? Have you experienced a stigma attached to Parkinson’s by other people?
Dancing	About dancing; how long have you been dancing? Can you remember a moment where you really enjoyed the dance class? Can you remember a moment where you didn’t enjoy the dance class? How does dancing make you feel overall? - Have you noticed any changes? What about other sports or exercise classes?/Are there any other things you find that help you with Parkinson’s?	What do you think of the dance for Parkinson’s classes? Have you noticed any changes in your partner since or when they have been attending dance classes? If so, please elaborate. Do you and your partner do any other forms of exercise together?
Relationship	How has Parkinson’s changed the relationship with your wife/husband? Can you remember a moment with your wife/husband that was very nice? Can you remember a moment with your partner that was difficult?	How has your relationship with your partner changed since they were diagnosed with Parkinson’s? Can you remember a moment in the past couple of months or so that you and your partner enjoyed? Can you remember a moment recently with your partner that was difficult? Have you noticed any changes in family dynamics since your partner was diagnosed with Parkinson’s? Do you ever feel angry with yourself or your partner as a result of their Parkinson’s? If so, can you elaborate on this? What do you think is the key to having a long and happy marriage?
Confidence/Self-Esteem	In the past couple of months or so, can you remember a moment where you felt proud of yourself for any reason?	Can you remember a moment where you felt proud of yourself for handling a difficult situation with your partner? Can you remember a moment in the past couple of months or so where you felt proud of your partner for any reason or dealing with a difficult situation?
Additional aspects	Is there anything else you would like to tell me about living with Parkinson’s or dancing?	Is there anything else you would like to tell me about life with Parkinson’s?
General Prompts	What was happening? How did it make you feel?	What was happening? How did you manage that? Imagine being in that moment, what was happening? How did it make you feel?

Since the interviews were semi structured, some of the topics brought up by the people with Parkinson’s were also included in the interviews with the care-partners, in order to get their thoughts on the same matters such as thoughts about the future.

### Procedure

Information about the study was distributed amongst participants of two dance classes for people with Parkinson’s by the dance instructors and the researcher. Only participants who registered their interest to volunteer as a couple were contacted and told more details about the study. The participants were asked not to discuss the questions with their partner until after both had been interviewed. The decision to interview them separately was made on the basis of wishing to gather data about each person’s subjective experiences and enabling them to share their experiences without having to worry about their partner’s view. The person with Parkinson’s was interviewed first (i.e., Participant A, C, E, G, I, and K). The time limit for each interview was 1 h, although the shortest only lasted 17 min (the participant had severe communication difficulties) whereas the longest lasted 50 min. The interviews took all place before the Covid-19 pandemic in public places such as a café, communal lounge, and a pub. A few participants had severe mobility impairments and were therefore interviewed in their home. All participants provided written informed consent prior to taking part in the interviews. After the interview, all participants were debriefed.

### Data Handling and Processing

The first author transcribed all interviews verbatim and pseudo-anonymised the data. Data analysis was conducted according to Braun and Clarke’s (2006) six-step thematic analysis. We followed an inductive approach as it was important to create themes from the data that cover the whole range of participants’ experiences, in and beyond dance. The first step, familiarisation with the data, started with transcribing all interviews followed by reading them several times and initial thoughts were noted down. In the second step, initial codes were generated, such as “dance instructors” and “needing help.” In step three these codes were then sorted into preliminary themes and sub-themes by writing them down and color coding the transcripts. Both authors independently conducted all steps from step 2 onward both authors and involved discussion until agreement on the codes was reached. In the fourth step the themes and subthemes were reviewed and adjusted. The final themes were created, reorganised, and written down in a thematic map and given definite names in the fifth step. This was followed by the final step, the report writing and revision. Although we aimed to understand the themes to cover the broad range of topics discussed, due to the word limit, the decision was made to discard some themes that appeared to not fit in with the whole data set or did not provide an answer to our questions.

## Findings

As the interviews were conducted and analyzed independently, the themes are reported separately as well (See Tables 2, 3). The inductive thematic analysis showed that disease-related thought processes take centre stage and emphasised that carers and their Parkinson’s partners share some experiences, the topics differ overall. A comparative discussion can be found in the discussion. To fully capture the lived experiences of dance for Parkinson’s, we included themes that are associated with but not directly “about” dance and/or their relationship.

**TABLE 2:** Themes from the interviews with people with Parkinson’s.

**Themes**	**Subthemes**
Changes–the Known and the Unknown	The mystery disease: Who is in control?
	Questioning the impact of dancing
	What others know or should know
Inclusivity and Connectivity	Seeing and being seen
	Social Enablers
Emotions	Positive and negative emotions
	Interactive Playfulness, incl. music

**TABLE 3:** Themes from the interviews with partners of those with Parkinson’s.

**Themes**	**Subthemes**
Responsibilities	Initiating and taking action
	Holding awareness
Openness	Ambivalence
	Social past and future
Opportunities	Coping
	Questioning the Effectiveness of Dance for Parkinson’s

### Parkinson’s Experience

#### Main Theme: Changes–the Known and the Unknown

Across all interviews, Parkinson’s participants described how they experience changes associated with their disease, including the symptom fluctuations, the impact it has on their relationships, as well as the potential positive changes through the dancing. What is particularly noticeable is that participants discuss changes within a framework of the known and the unknown related to the mystery disease. This is of interest considering that with its powerful yet intangible experiences, mystery is a strong characteristic of dance.

##### Sub-theme: the mystery disease

Participant K illustrates the gravity of changes associated with Parkinson’s: “*It means a complete change of the way of life*” and “*this is going to get worse*.” Overall, the direction of the disease is clear, and all participants report on the things they are not able to do anymore. Yet above all, participants’ responses illuminate how several changes are based on “unknowns” of the disease:

“*Well when you receive the diagnosis you don’t know what it means, you just don’t know what it means. And you start learning fast*” (Participant K).

Other unknowns of the disease are the unexplained symptom fluctuations, which make short- and long-term planning with the progressive disease difficult. Participant C states: “*So my life is completely unpredictable uh*… *it suddenly changes and I’m not good uhm*.” The unknowns of the disease are also present in the experiences our participants had in receiving a diagnosis. Participant G, for example, explains:

“*No, he (the doctor) didn’t pick up on it for a while, he just said that we’re watching the symptoms. It’s quite hard to diagnose*.”

Participant I’s doctor wasn’t sure either as he explains:

“*And I went and see him [doctor] and he said yeah you’ve got Parkinson’s I think*…. *what does that mean? Does it mean I’ve definitely got it or I haven’t got it, he said well they don’t know we’ll have to see*.”

Once diagnosed, the next step is generally the medication, which can be continuously changing in type and frequency. On some days, participants require more pills, which participant K explained “*it’s uh a pain*… *It comes to dominate your life*.” Moreover, whether the medication helps or not is not necessarily known, as Participant A says:

“*The whole process of medication as far as I’m concerned, I am not conscious of it doing any good. But neither am I conscious of it doing anything wrong. Erm maybe it is that I am expecting miracles and not getting them or I don’t know*.”

This is of interest as whilst most participants with Parkinson’s question the impact of dance also, they nevertheless experience it as doing them good (see sub-theme below). Participant A further illustrates the challenge for them to situate their condition as noted in the dancing with humour:

“*And some people in the class say that if I forget to take my medicine, I’m in a terrible state*…. *(Pause) Maybe I am in a terrible state but I think it’s my normal state (laughs) I don’t know*.”

It is possible that the unknown associated with Parkinson’s is one of the reasons as to why comparisons with others are so important as discussed under the separate main sub-theme “seeing and being seen.” Clearly, the direct impact of Parkinson’s is substantial and conscious not only in their dancing but their everyday behavior as well as during the interview. For example, participant K was apologetic when struggling to answer a question: “*Ehm (whispers to themselves) sorry this is not working out very well*.” Considering that much is unknown about Parkinson’s whilst participants are aware of their struggles, it is not surprising that they create their own theories. In response to a question that was unrelated to the causes of the disease, participant C lists possible reasons such as concussions experienced as a schoolboy. Also, participant E illustrates the links to anxiety as follows:

“*Yeah I, I’ve always said it’s a vicious circle, the anxiety will feed the Parkinson’s and the Parkinson’s because of the symptoms will make you anxious so it’s just, vicious circle*.”

Yet at the same time, participants know what is recommended to help with the symptoms, one of which is dancing. In most cases, they state that they are compliantly adhering to these as is with this participant:

“*And I just think that some of the ways of trying to break through that is through stretching, or exercise, or your diet, whatever, to try and break that circle. Yeah that’s why I’m still doing ballet after 3 years yeah cos it’s good for me so*.”

##### Sub-theme: questioning the impact of dancing

Participants discussed changes in relation to dance participation throughout the interviews. When participant E for example talks about his lost ability of writing or engaging in other daily activities that require fine motor control, they refer independently to the ballet classes:

“*Yeah uh I lost the ability to write properly and stir a cup of tea*… *it’s completely in slow motion and I was looking at my hand and just thinking my god and I get frustrated*,… *and when I, ballet, we used to do this with our fingers (shows finger exercise) I was just like this really slow and I thought I’m dying, but through this ballet and other things that I do I’ve got all of this back, yeah I can stir a cup of tea, all of this has come back and yeah*.”

Notably though, according to participant E, the feeling of control returned due to ballet and “other things.” Indeed, not all participants were convinced that the dance classes help on a physical level. Participant C for example notices changes but likewise questions the impact of dancing:

“*I think I’m walking a lot better for whatever reason I don’t know*… *whether the um classes were helping I don’t know, I suspect they were*.”

What remains unquestioned across the Parkinson’s participants, however, is that dance is good because it provides a change in their environment. It gets them out and includes social contact as discussed in the sub-theme “social enablers.” Moreover, it is clear from the interviews that dance evokes overall more positive emotions, as discussed in the “emotion” themes. Yet, for dance to be enjoyable, participants expressed that the dance-classes need to make sense. The classes need to be able to relate to the participants and their conditions as also discussed in the following sub-theme. It helps when participants are able to relate the dancing activity to something they know or can understand. For example, participant C describes that they could not execute gestures they didn’t know or didn’t understand:

“*I couldn’t relate to it, she it had, it had a story line that was to me incoherent, it didn’t make sense. And we were making gestures like grass sunshine or something um and I can’t remember what they were but they were sort of random concepts which to me didn’t make any sense and uh I found myself sort of unable to follow because they were un, to me they were not, not sequential and logical. Um I don’t know what else, I didn’t enjoy that. I couldn’t actually I couldn’t do it really*.”

The same participant also illustrated an example of gestures they enjoyed as they knew them from their travels abroad:

“*well I love [the dance instructor’s] gestures and I love the, the, and the first time she introduced the uh the gesture of this (does twisting movement with hand) cos I used to work in Italy*,… *And I remember all sorts of gestures that they had*…”

Nonetheless, participants also experience the dance classes as enjoyable when it surprises them, as discussed in the section “Sub-theme: Interactive Playfulness.” Thus, the impact of dancing is situated between the known and the unknown, as is the disease.

##### Sub-theme: what others know or should know

Further changes that the participants emphasised were connected to relationship dynamics. Changes with their care-partners were not experienced as sudden; but it was also emphasised that they weren’t planned. For example, participant K explains:

“*Well I’ve accepted that things are not the same as they once were ehm and that she’s, we’ve developed into various roles. She looks after me which wasn’t the original contract*.”

However, it is noticeable that these changes weren’t discussed in much detail. For example, participant G mentions changes but they are not comfortable to elaborate on these neither with the interviewer nor their partner: “*Uh (long pause) communication, uh the sexual side I suppose*,… *I just keep it to myself*…. *nothing you can do about it*.” Or participant I “*Sexually it, it has done really we don’t (mumbles)*.”

It is evident in the interviews that participants recognise that their care-partners “know” more and are more in control, as participant A explains: “*[their partner] actually stop me from doing things that I think I’m still capable of and it was a bit of a frustration*.” This is further elaborated in that their partner could see things which they themselves couldn’t: “*Not that I noticed, but my wife did notice*… *when the diagnosis first came through, she said oh yes I recognise that sign*” which also links to the sub-theme of seeing and being seen.

The participants also illustrate how important it is for them that others know about their condition not just for their everyday life encounters, but also for dance classes. Participant E reports several distressing shopping experiences, where staff and other shoppers didn’t know how to respond kindly. Dance examples are discussed in more detail in the theme “Inclusivity and Connectivity.”

#### Main Theme: Inclusivity and Connectivity

Inclusivity and connectivity are important topics that were explored several times by the Parkinson’s participants in relation to their everyday life as well as the dancing. Seeing and being seen as well as having social contact through what we call “social enablers” constitute relevant parts of their experiences.

##### Sub-theme: seeing and being seen

It is interesting how participants related to the importance of seeing. This ranges from seeing others’ developments as well as seeing nice things. For example, “*having the grandkids coming along you know*… *has really changed things because I need to survive to, to watch them grow up*” (Participant E). Observing changes gives participants a purpose of continuation and seeing natures’ beauty can keep them interested in an activity:

“*We have a place in the mountains in France*… *and the um one of the objectives of going there is to facilitate the interest in walks. So there are we’re very near the mount blanc and so you get to see the spectacular snow cap*” (Participant A).

Yet, seeing is also of particular interest since it can lead to struggles associated with the disease, such as bouts of freezing:

“*me with going into Sainsbury’s and Robert Dyers and places like that, when I see it’s full lots of items about, I just can’t do it. You know my brain says no, stop, or I’m going to shuffle you into something so*” (Participant E).

Similarly, seeing thresholds is a challenge: “…*or stuck especially under doorways. Um I have great trouble going through thresholds*” (Participant C). Thus, albeit not mentioned, the environment of dance classes which are in relatively large, uncluttered spaces seems optimal (but see also section “Sub-theme: Interactive Playfulness”).

Moreover, when Parkinson’s symptoms are visible from the outside, participants can experience judgemental responses from others. For example, when other people see their symptoms and assume, they are drunk. Or how participant G observed: “*I notice when you come into a room people show away from you*,” which was not mentioned to happen in the dancing. On the contrary, participants are passionate about their dance related achievements. For example, one participant told their daughter proudly that they have taken on a leadership role in the dance classes twice, despite their daughter’s downplaying response: “*But it’s ballet!*”. Instead of letting themselves being affected by the social stigma attached to dance, it is evident that being seen by others through dance can provide participants with an opportunity to experience pride and confirmation, as illustrated by participant C:

“*yeah*…. *it was a show for you know, 300 people*…. *um I went on stage to do a [solo], 15 min of um my chaotic dance models*… *But I was a bit nervous that I, I couldn’t hold a stage anymore but it, it was quite good*”

Notably, participants also report taking confidence from other forms of public presentations. Since there are many unknowns of the disease, being seen by others and seeing others can help Parkinson’s participants to situate themselves. Interestingly, the observation of others with Parkinson’s during the dancing is mentioned rarely (see example sub-theme positive and negative emotions). Observing and comparing oneself with others is however an integral part of their everyday life, mentioned in contexts such as sitting in the doctors’ waiting room or watching TV. Clearly Parkinson’s dancers also observe the instructor (see section “Sub-theme: Interactive Playfulness”) and being seen as a person with Parkinson’s in the dance classes is relevant. Overall, it is very important for participants to “*getting some degree of confidence and self-awareness where one is at for real and where one may not be*.” (Participant A). This is also evident when participant E talks about a class that he didn’t enjoy at all:

“*and the instructor said to me what’s wrong and I said well it’s just we’ve got uh this disorder and he has quite clearly not looked into it because the stuff he was doing people can’t do that and I myself struggled a bit and then but the instructor took it onboard and um he never came back and we’ve never had that since so yeah*”

As the symptoms of Parkinson’s are not necessarily seen from the outside as explained by Participant E, responding to the limitations can be a challenge for others: “*as I’m sitting here now and I look fine, inside I can feel myself doing this kind of movement (shows swaying in circles movement)*.”

##### Sub-theme: social enablers

Social enablers are activities or people that allow our Parkinson’s participants to feel part of something, supporting a sense of inclusivity and connectivity. Dance was mentioned as a social enabler throughout the interviews. Participant E states “*Yeah uhm well I’ve been there a long time now so I feel very much a part of it*.” Participant G, for example, describes a particularly enjoyable aspect of the dance classes:

“*Being able to move about without the fear of falling down and the contact of people*… *to social side is good, yeah*.”

Another way in which the dance classes are experienced to achieve a sense of connection is through the music:

“*I think quite good and there’s some classical music but there’s also some ehm popular songs including my generation so people like the Beatles, it incorporates some of these musicians into ehm into distraction and it’s entertaining actually*.” (Participant A).

Other participants also refer to the importance of the music relating to their group in positive and negative ways:

“*Um I’m quite happy with it (music), people are mostly my kind of age so the music is appropriate, year and quality, like the Beatles for instance*” (Participant K)

“*I’ve enjoyed it (dancing) a lot every week but about a year ago they brought a young lad (instructor) in that was doing his own type of music and dance and he lost everybody. Everybody was just sitting and just didn’t join in in it and I got very angry about*” (Participant E)

It is interesting to note in the above examples how Parkinson’s participants often extent their own experiences to the whole group, which could be read as a sign that they do feel part of the group. Notably, the caring for each other goes beyond the disease or the dancing, as explained by one Parkinson’s participant that started language lessons to improve their Dutch through a contact from the dance classes.

Nevertheless, it is important to remember that dance classes are not the only social enablers noted to be of relevance for Parkinson’s participants’ wellbeing. Notably, the connections to family members beyond their care-partners are often mentioned as particularly enjoyable. For example, one participant very much enjoyed their wedding anniversary with the whole family. Moreover, whilst participants enjoy their family looking after them, this is also recognised as giving their care-partners a respite from care.

#### Main Theme: Emotions

##### Sub-theme: positive and negative emotions

Parkinson’s participants refer to a range of positive and negative emotions throughout the interviews. Whilst the expressed emotions are predominantly negative in relation to the disease, such as anger, lack of motivation, embarrassment, frustration, depression, or resignation, they are mostly positive in response to the dancing, such as happy, fun, enjoyment, motivating, encouraging, liberating, or a feeling of confidence. What is noticeable is that participants name a multitude of factors that make dancing such a positive experience. For example, participant A explains: “*I find I find dancing is not a natural activity for me. But, if you combine it with a bit of music it can become fun!*” Similarly, as participant K describes: “*Well it’s a social event, which one doesn’t have many of these days. Uhm and it’s exercise and moving to music so it’s fun*.” In this statement, the participant names all the relevant facets of dancing, which are social, emotional, physical, and auditory. While participant C specifically refers to the movement aspect as providing them with a sense of liberation:

“*based upon my, my mask like lack of expression and um my gait, and my not swinging the arms particularly well*…. *Which is one reason that I, I always think about that when we do these exercises in class, it’s very good. Specially the twisting of the body because some in the class are doing that (shows poor twisting movement) but actually what the instructor is doing is that (does huge twisting of the body) and it feels so, so good so liberating*.”

Similar to the sub-theme of social enablers, where dance is an important but not the sole source for social contact, participants have positive experiences beyond the dancing through other activities, such as walking, or going to events. For example, the Parkinson’s participant K very much enjoyed an event with their care-partner at the British Library: “*well we went for something unusual, we went to a lecture at the British Library, which we both enjoyed I think*.”

Moreover, dancing might evoke a mix of emotions. For participant I, attending as a couple was the main reason for one of our participants to attend:

“*I can’t say I really enjoy it, I don’t mind doing it, I quite, quite enjoy it. I’ve never been uh I’ve always been athletic but never been dancing. I was (mumbles)*…., *I’m not a dancer (laughs) ‘they’ (care-partner) are a good dancer*…. *I just come because of ‘them’ (care-partner) because they want me to keep dancing once a week and so we come here*.”

##### Sub-theme: interactive playfulness

A particular positive emotion expression that shines through all interviews with the Parkinson’s participants is their sense of humour and enjoyment in playful interactions. It seems that participants with Parkinson’s like to be surprised, despite all the uncertainties they face caused by their disease. Participant E describes the joy of new routines and humour:

“*the instructor I think is unbelievable, the amount of different things that ‘they’ know*… *and the one thing will just trigger it off, you know one word and then ‘they’ll’ ‘they’ll’ ‘they’ got something to make from it, ‘they’ are incredible*.” And later: “*It suits me but uh I can imagine somebody saying that ‘their’ sessions are too random and illogical but um but we share the same sense of humour*.”

Even those participants who might not enjoy dancing as much use a sense of humour and playfulness to keep doing it: “*I do it and I make it, make it enjoyable, and having a laugh as well with it, don’t take it too seriously you know*” (Participant I). This participant later adds: “*I’m never going to be in Phantom of the Opera (laughs)*.” A reference to the importance of interactive playfulness can be seen in Participant A’s a detailed analysis of why they find it easier to walk on uneven surfaces:

“*for walking, it’s sometimes easier for me to be walking on difficult land, difficult ground, than it is on straight ground um straight, flat, ground. If you gave me the choice between walking across an air terminal on a completely even featureless um concrete or slab flooring, um versus boulda hopping as it were a mountain, on lake district mountain, were you have to sort of go in another way and*… *which*…. *you’re going to step on. I find that easier than walking across a featureless floor*”

Notably, dance spaces are flat and often featureless. Thus, this experience is in opposition to the previous cited examples of the problems Parkinson’s sufferers have with thresholds and visual information. Participant A goes on to explain:

“*Because I am applying the active cognitive sort of thinking part of my brain rather than the sort of primitive um sort of primitive low the um the primitive brain which is the automatic. So if I were to walk across a completely featureless*…*um and to do so if I try and do so and to get into a rhythm I find that once I have consigned it to my automatic walking the little*…. *and I will sort of get into a stuttering movement, but if I am*… *everything has to be controlled by the deep thinking part of the brain, to step or not, sort of working it out, so for that very good reason it is easier because it’s not being automaticed, automised, yeah*.”

Whilst this explanation contradicts what we know about motor control (i.e., in that it is the difficult terrain will activate more subconscious processing, by removing the cognitive conscious control of the individual), it is interesting as it emphasises the ways in which an element of interactive playfulness in movement can facilitate motor execution for people with Parkinson’s. The playfulness in this example is stimulated by the environment, whereas in dance it is through the imagination, the music, or the group interactions.

A sense of humour and enjoyment with interactive play is not only present for the dancing. For example, the Parkinson’s participants who have the opportunity, love to play with their grandchildren. The risk involved in playing with grandchildren is however evident and sometimes, if the Parkinson’s is too restrictive for physical play, this can create a sense of loss:

“*We have grandchildren, and that helps too when we can get to see them. It’s fun to catch up with them but even there Parkinson’s has a negative effect because I can’t play as vigorously with the children the grandchildren which I would like to do but I can’t*” (Participant A)

Whilst the dance instructors can adapt the classes to their group, playing with grandchildren is less flexible. Also, it exemplifies that beneath the humour lies something more troublesome. Participants with Parkinson’s know some things are beyond their control and humour can be a means to build resilience. Participant K for examples responses to the closing question whether there anything else they would like to add about living with Parkinson’s:

“*I cannot recommend it, don’t write that down (laughs). (Mumbles) people don’t always appreciate it. Um no, I’m extremely lucky to have ‘them’ (care-partner). Without ‘them’ I don’t know, I’d probably be in a home somewhere (pause)*.”

### Care-Partners’ Experience

#### Main Theme: Responsibilities

##### Sub-theme: initiating and taking action

In many ways a Parkinson’s diagnosis brings a lack of and a need for control into a couple’s life. Since the diagnosis is multifaceted and impacts the person’s symptom severity, responsibility is put on the partner to always be prepared for administering booster pills or help preventing falls. This significantly restricts what they are able to do as a couple on a daily basis. It also places care-partners in the position to impose or be in charge over big and small decisions, such as a house move or which transport to use in prediction of their partners illness progression and symptom fluctuations. These everyday management tasks also appear in regard to the dancing. For example, most care-partners said that they took the initiative to start going to dance classes, and without their prompting and encouragement, their partners would not attend. As participant J reports:

“*We, we like it. We have to, I have to drag them here, they probably told you*… *they used to play a lot of football but they’ve never been into exercise*.”

The care-partner therefore often has a lot of responsibilities related to their partner’s daily activities, ranging from attending dance classes to having a medication review, all of which can take a toll on their wellbeing because it demands non-stop attention. Considering the carers responsibilities associated with these everyday challenges, attending dance classes cannot be understood in isolation.

##### Sub-theme: holding awareness

In cases where the Parkinson’s is quite progressed, it increasingly becomes the carer’s responsibility to continuously pay attention to the wellbeing of their partner. It is of importance to note that the continuous awareness and guidance is a theme for care-partners outside dance classes but not within. For example, carers need to know when to give so called booster pills to help manage the Parkinson’s symptoms, but even then, their partner may still go into “off” mode and shut down, such as participant J states:

“*‘they’ are having a bit of a bad day today really with walking so we’re going to have to wait and see how ‘they’ feel but ‘they’ might be alright when we leave here (to go to the dance for Parkinson’s class)*.”

This unpredictability means that many care-partners give up some of their interests and other responsibilities such as volunteering, in order to support their partner. Participant H commented:

“*Eh as far as I’m concerned it’s obviously meant that I’ve had to increasingly care for ‘them’ and give up some of the things that I used to enjoy doing so that I can look after ‘them’ and that’s been increasingly the case as the Parkinson’s has developed*.”

Thus forth, the dancing offers care-partners an opportunity to combine their own interests with the needs of their Parkinson’s partner as well as the ability to take away some of the carer’s everyday responsibilities through a reduction in the otherwise ceaseless attention and awareness they have to dedicate to their Parkinson’s partners. In general, care-partners try to promote their partner’s independence as much as possible but sometimes this means more work for them if not everything goes according to plan. Notably, two of the care-partners did not attend the dance for Parkinson’s classes with their partners and were not involved in the dance programme in the same way:

“*I can probably think of one, maybe two, that I’ve taken ‘them’ (Parkinson’s partner) in the car, um which hasn’t usually been because ‘they’ve’ been incapable of getting under ‘their’ own esteem it’s usually because it’s late. So I don’t really have the experience of ‘them’ going to the class*” (participant D).

They both felt that they did not need to support their partner in going to the classes as they were mentally and physically able to make their own way there and back. Importantly, the carers also reported that they used that time for themselves. The dance classes are thus an opportunity to reduce the care burden, whether by attend together as a couple or not (see also section “Main Theme: Opportunities”).

#### Main Theme: Openness

##### Sub-theme: ambivalence

Openness is not only important in the relationship for communication purposes but can also be found within the dance for Parkinson’s classes. The care-partners had found a safe, support network within the dancing as they were all in the same (or a similar) situation and had a level of understanding of what the others were going through. One participant had noticed that her partner opened up at the dance classes and commented:

“*And there’s this you know when you meet somebody, when ‘they’ meet people and ‘they’ chat ‘they’ have this energy but on ‘their’ own or with me it’s calmer and it’s not the same*” (Participant B).

This caused somewhat of a conflict within the participant, as they on one hand were happy that their partner had this extra energy and was very open and sociable when at the classes, but on the other hand they said that their partner did not behave or feel that way more often or when it was just the two of them at home.

The care-partners seemed to have largely resigned to this new way of life where their partner had Parkinson’s and all of the changes that it had brought upon them as a couple. Participant L also said:

“*I say look in the mirror you know, if you have to do that (reach arm up) you do that (barely lifts arms) and ‘they’ just doesn’t feel the difference, ‘they’ feel that ‘they’ have had a good workout, if ‘they’ shrug ‘their’ shoulders and you can barely see it! I uh I think it’s good that ‘they’ go out and try and ‘they’ say that ‘they’ do ‘their’ best so, so I can’t, who am I to say I’m sure ‘they’ could do better*.”

This quote illustrates how the care-partner has resigned to their current situation even though they would like them to try more. Hence, whilst a level of openness allows care-partners to engage in their partners wellbeing, there are levels of criticism involved. Many talked about accepting the situation although most of them mentioned feeling frustrated, sad, disappointed, or angry at times, not directly at their partner but at the situation or Parkinson’s diagnosis and not being able to express this and instead carry it within them. Participant J commented “*I just worry sick every day*” and added:

“*I think they gets frustrated cos we sit here, we sit there, and think how did we end up here? You know what I mean? After the life we’ve led, here we are*.”

##### Sub-theme: social past and future

The care-partners who regularly attended the dance classes said that they enjoyed being able to attend a social event together, as this is something that they are unable to do as much these days. The dance classes can give the couples an opportunity to both interact and extend their social networks together as a unit

“*we always enjoy the exercises and the chat that goes to and fro and it’s a, you know a bit of a social occasion as well as exercises, you’re seeing the other people*” (Participant H).

However, for some people with Parkinson’s it may be difficult to socialise and make new connections due to the cognitive and verbal challenges of Parkinson’s, something which participant L had noted

“*they (‘their’ siblings) really take ‘them’ out of ‘themselves’ in that because it’s so familiar ‘their’ brain goes into different gear when ‘they’ speak to them and I find with old friends as well. More so than with people we’ve only known for the last few years but ‘they’ are more confident and ‘their’ brain works better. ‘They’ remember more because ‘they’ are beginning to be forgetful and remember more about the past*.”

There is often a change in the dynamics or roles within a relationship after a Parkinson’s diagnosis as the healthy partners need to do more either physical chores or cognitive tasks, depending on in what ways their partner has been affected by Parkinson’s. However, the care-partners noted that the care burden is not something that occurs overnight, rather it accumulates slowly over time. Participant D said

“*I think the, the difficulty for me has been mostly not knowing how ‘they’ are feeling, managing, um ‘they,’ ‘they’ show ‘their’ feelings but ‘they’ must have feelings and anxieties you know long-term and it worries me*”

and participant L added “*It goes so slowly and the tasks and the burden becomes heavier and heavier, you get used to*.” This was something most care-partners refrained from sharing with their family and friends, as they did not want to worry anyone else and therefore carry this burden by themselves.

#### Main Theme: Opportunities

Some of the care-partners mentioned how they benefitted from the classes as several of them said they enjoyed dancing and rhythm came naturally to them. They talked about how they have danced throughout their lives, although not together with their partners who had mainly played various sports previously. The dancing thus offers itself as an opportunity for carers to do something with their partner that they enjoy doing. This is pertinent, as other activities caregivers enjoyed are challenged through the attention their partners need. For example, several carers described how they enjoyed playing with grandchildren, however, they were not able to fully engage in these activities as they needed to always divert a lot of attention to their Parkinson’s partners. Henceforth, it seems that the dancing is an activity in a controlled environment that allows care-partners to be more be present in themselves. In addition, it also provides an opportunity to gain a better understanding through social comparisons with people who are exposed to a similar situation as them.

##### Sub-theme: coping

The care-partners have a unique outlook on Parkinson’s as they spend most of their time supporting their partner compared to family members or friends who may only see them for a few hours now and again. Some care-partners noted that their children did not understand the severity of the situation, but at the same time other care-partners were very apprehensive about sharing too many details with their other loved ones as they either did not understand or worried a lot. They also talked about how the general public does not have a good understanding of Parkinson’s and had experienced some judgemental looks whilst out and about “*they look at you as if you’re drunk! You know, falling all over the place*” (Participant F).

For some people the dance classes were thus an opportunity to discuss with other care-partners what they had gone through in the past week and compare experiences. Participant B commented

“*Because precisely we see quite a lot of people and we can compare whereas before if you do not see others you’re quite isolated but there you can see how people evolve and so you are not on your own and so I think that has helped them and me to see the range*.”

She spoke about feeling isolated and lonely before attending the dance classes. Participant H felt very much the same way, and said

“*You know that’s, that’s one of the good things about the group, from the point of view that uh whether it’s exercises or the group itself for people on their own, at least they can, they realise that they’re not on their own, other people have got it too and they’re all coping and we’ll have a chat about it, how you manage and ideas have come up about doing different things, yeah*.”

One participant did however point out that it is impossible to know how Parkinson’s is going to affect their partner further down the line and that it is pointless to compare them to others since everyone gets different symptoms.

##### Sub-theme: questioning the effectiveness of dance for Parkinson’s

The care-partners also questioned the dance classes to some degree regarding motor improvements with most of them saying that they did not notice any motor related differences in their partners after beginning to attend the dance classes. They did not talk a huge deal about the physiological effects of dance, although participant D did comment “*so I can’t tell really physically, if ‘they’ve’ walked there and walked back, it’s difficult to quantify*.” As their partner walks to the classes and keeps active outside of the classes too, the care-partner felt unable to determine any associations between dance and physical abilities as those may have been influenced by the walking.

The role of music was not mentioned as having a specific impact either, with one participant saying that the music does not do anything for their partner whatsoever “*Uhm I’m not sure, ‘they’ have no sense of rhythm, so the music doesn’t do anything for ‘them’*” (participant L). Only one participant explicitly stated that they thought the music alongside the movements were beneficial for their partner:

“*I think it’s very good I think uh (pause) the way of using movement and dance no movement and music, movement and music whether it’s dance or not (laughs) it’s really excellent for the brain because the brain makes the connection and it’s very good for its agility*” (Participant B).

## Discussion

Parkinson’s is often described as a mystery disease since the prevention of the disease is unknown, the causes of non-motor symptoms not fully understood, and its progressive symptomatic individual and varied (e.g., Ball et al., 2019). This mystery characteristic of the disease is specifically visible in the interview responses from people with Parkinson’s: The unknowns associated with the changes that the disease brings were the most frequently discussed theme across all interviews but also played a role across other themes, such as seeing and being seen. Since Parkinson’s participants often expressed that they did not have a clear sense of where they are in the progression of their disease, they compare themselves to others. Seeing others with the disease in everyday life as well as in the dance classes allows participants to reduce the level of the unknown. Since the Parkinson’s sufferers themselves and their doctors are in the unknown, then, who knows?

Therefore, the question “Who is in control?” overarches the theme of changes and its associated knows and unknowns. In a way, seeing becomes a way of knowing. Also, having a care-partner who is in control is another important pillar. Whilst participants recognised this, they did not seem to be at ease to share relevant information with either partner or others, such as the interviewer. One possible reason is that communication is an overarching challenge for Parkinson’s sufferers (Saldert and Bauer, 2017). Nevertheless, it is important for people with Parkinson’s that other people know about their condition. This is particularly evident when they described challenging everyday experiences, but also when they talked about the dance classes. This is in line with the findings by Rocha et al. (2017), which showed that dance instructors need to be experienced and able to modify and adapt the dance classes to the participants’ needs for them to enjoy dancing. Instructors that run classes beyond the participants’ abilities were explicitly not welcomed by our participants.

Notably, the unknowns also occurred within the responses of their care-partners. In response to the question of “who is in control?”, where possible, the partners take over. For example, in most of the cases, the care-partners were the ones who took the initiative to attend the dance classes together due to several reasons such as wishing to encourage physical activity, socialising, or to do an activity together without being restricted by the Parkinson’s diagnosis. The socialising and support aspect were important to many care-partners and is something that has been found in previous studies too, such as Rocha et al. (2017), who found that some participants attend classes mainly for the social support. This was echoed by the current study, as the care-partners felt it was beneficial for both themselves and their partner to meet and spend time with others with Parkinson’s as everyone was in the same situation. The dance groups can create excellent support networks where everyone gets to feel understood and valued, as well as getting guidance how to live life with Parkinson’s (Houston and McGill, 2013; Kunkel et al., 2017). This is particularly important as the care-partners often felt as though they were unable to tell their friends and family about the severity of the situation and their partner’s Parkinson’s progression.

The social aspects of the dance classes also create opportunities for connections beyond dancing or Parkinson’s, giving care-partners a potential respite from their care burden and for the romantic relationships to enliven at the same time through other external stimulants. This is important since with the progression of their partners’ Parkinson’s, the care-partners take on an increasing amount of responsibilities which can lead to an increased care burden and decrease in mental wellbeing. However, both the people with Parkinson’s and the care-partners noted that this progression happens slowly and whilst a change in the relationship dynamic with the care-partner taking on chores they haven’t had to previously is unavoidable, adapting to the changes in responsibility and control is crucial. Nevertheless, the unknowns encountered by the people with Parkinson’s were mirrored by the care-partners. Care-partners struggled to come to terms with not knowing how Parkinson’s might affect their partner in the future, whether they need to move, employ a live-in carer, or find a place for their partner in a residential home. Due to the unpredictability of Parkinson’s it also often means that care-partners have to give up activities they used to do as they were unable to leave their partner alone at home. Whilst previous research found that changes in the relationship dynamics can occur drastically after one partner is diagnosed with Parkinson’s and the other one slowly becomes a care-partner (Karlstedt et al., 2019) and these changes are evident in our data, they are not the sole factor of concern.

The care-partners spoke to varying degrees about the potential physical benefits their partners may notice through the dancing, although one care-partner in particular pointed out that her husband was not aware of not moving his body properly. A lack of self-awareness is a common consequence of Parkinson’s (Maier et al., 2012) and is what Haahr et al. (2011) found as those with Parkinson’s often perceive their bodies as not functioning properly, for example limbs not following orders. Interestingly, negative body perception is frequently reported to affect levels of wellbeing in Parkinson’s (Hadley et al., 2020), but none of our participants expressed specific suffering with their body image or appearance. Hadley et al. (2020) noted, however, a lesser increase in bodily awareness in people with Parkinson’s who took part in dance classes compared to a matched control group. The authors also observed that whilst dance can lead to an increased bodily awareness, this includes the awareness of body limitations. Therefore, our care-partners might experience similar frustrations, in that the dance participation highlights their partners’ limitations. Accepting the changes, restrictions, and adaptations appear to be a vital part of living a happy and fulfilled life with Parkinson’s, not only for the person themselves but for their partner to cope as well (Phillips, 2006).

Interestingly enough, both participant groups were rather critical of the facilitating role of music on movement. People with Parkinson’s found that the music highlighted what they could not do and that it challenged the understanding of the instructions, due to the increased level of volume, a common observation for people with Parkinson’s (e.g., Mollaei et al., 2013). However, the choice of music was considered an imperative part of the overall dance experience for people with Parkinson’s, possibly as it allowed them to reminisce and reminded them of their youth. The music could make them feel recognised when it suited to their generation, their experiences, and their preferences, creating positive emotions. Rocha et al. (2017) also found that the choice music is important to enhance memories, thus increasing the emotional and social benefit of the dance classes.

Overall, both the care-partners and those with Parkinson’s questioned the impact dancing may or may not have on any physiological symptoms. Whilst it is evident through an extensive number of qualitative studies that dance for people with Parkinson’s is beneficial for their emotional, social, and psychological wellbeing, quantitative findings on motor improvements are mixed (Jola, 2020) with at times evidence for better improvement trough other types of exercises (Rawson et al., 2019). Importantly, since we completed our interviews, two other studies were published that included care-partners in the data collection and analysis (Prado et al., 2020; Prieto et al., 2021). The study by Prado et al. (2020) is a mixed-method study that investigated whether psychosocial interventions, such as dance classes, have a positive impact on the wellbeing of people with Parkinson’s and their carers and what the motivations of carers are in either attending or abstaining from these sessions. The study found that relational, responsibility and uplifting elements are of importance for carers to accompany individuals to the sessions. Whilst these are similar with some of our observations, we would refrain from a direct comparison since the study focussed more on the meaning of wellbeing for carers and included interventions other than dance (e.g., music, swimming). Moreover, in the quantitative evaluation, the authors did not find a significant relationship between higher levels of wellbeing of caregivers or people with Parkinson’s and their joint participation in psychosocial activities, nor did the authors find a significant relationship between the type of physical activity and form of improvements (i.e., social or physical).

The findings of the most recent dance study involving Parkinson’s carers by Prieto et al. (2021) correspond partially with ours. These are in regards to the social relevance of the dance classes. Notably, the self-evaluation theme of people with Parkinson’s ties in with our themes of seeing and being seen, and dance as a social enabler. Moreover, we did have some indications of Parkinson’s people putting effort into complying to keep moving as dance might not feel naturally to them. However, we did not identify keep moving’ as a theme, as all but one of our Parkinson’s participants have always been physically active and overall expressed to enjoy doing so. It is important to note that whilst Pietro et al. (2021) distinguished the experiences of carers and people with Parkinson’s, it is not clear whether the interviews as well as data analysis were done independently for the two groups or not. Importantly, we conducted our data collection and data analysis separately for people with Parkinson’s and their care-partners to target individual experiences. Moreover, we only interviewed lifelong couples whereas the participants in Prieto et al. (2021) study included friends and other relatives as care-partners. Finally, the questions by Prieto et al. (2021) directly addressed the dance experiences; whereas we made the conscious decision to provide participants with the opportunity to report their dance experiences more freely whilst also focussing on aspects of their relationship which can explain the differences in our findings. We argue that a more open format allows participants to feel confident in questioning the impact of dance, which is one of the main differences between our findings and theirs.

We suggest that the questioning of the efficacy of dance classes by our Parkinson’s participants and care-partners is at least partly based in their extensive experiences of the many unknowns associated with the disease. Participants with Parkinson’s showed several attempts to solve the mystery of the disease, hence, they seem to have become predetermined in questioning any of their observations. It is important to note that, indeed, one participant was able to pinpoint a particular movement in class that provide them with a sense of liberation in their range of motion. Moreover, another participant reported how they were able to transfer a particular skill trained in the dance class to their everyday life. Whilst dance might emphasise the motor limitations of the Parkinson’s person in their care-partner, this might not have the same impact on the person with Parkinson’s. For them, it is important how something feels internally. And other research with stroke patients, another neurodegenerative disease with motor-impairment, has shown, that it can be the sense of achievement rather than the actual motor execution in movement training that is of importance for the individual and consequently the improvement (Grabherr et al., 2015). In other words, from the perspective of the person with Parkinson’s, imagined motor executions can be sufficiently motivating. As Grabherr et al. (2015) noted that they had stroke patients who reported feeling very comfortable with the motor imagery training due to an enhanced sense of control and the ability to “do something”. Moreover, our findings are in support of the suggested framework by Bek et al. (2020) in how internal action representations may contribute to the beneficial effects of dance in people with Parkinson’s. For example, we found that for at least one participant, movements in the dance classes could be transferred to everyday life contexts. For such an action correspondence to be consciously recognised, motor imagery in everyday life is necessary. It is thus possible that the dance training increases motor imagery abilities and thus contributes to transference of dance from the studio to everyday life. It is also of interest to note that observing others is a relevant theme for our Parkinson’s participants as is how it feels internally; both often assumed to be associated with imagery processes. We don’t know, however, whether observing others is a predominantly social or action related process. Interestingly, though, our participants did not report that the music would affect their motor representation or motor execution skills as suggested by Bek et al. (2020).

Finally, four of our six couples attended dance classes together. We neither observed evidence for a difference in our participants’ experience in response to dancing together or not. This is surprising considering the findings by Hackney and Earhart (2010) as well as Kunkel et al. (2017), who observed a bigger impact for Parkinson’s sufferers when they attended Tango classes with their partners. One possible explanation is that these effects are specific for Tango, a dance style whereby partners communicate through subtle weight shifts, thus increasing the intimate sensory attention of each partner, whereas the dance classes our participants attended consisted of a mix of dance styles. Further research is required to explore the impact of the dance style on the relationship. We suggest, however, two alternative explanations for this effect: Firstly, as indicated above, participants did not seem to feel comfortable in sharing their very personal experiences neither with one-another nor the interviewer. It is therefore possible, that communication issues are already present that are not alleviated through the dancing, or at least not in a way that it could have been shared with the interviewer. Secondly, it is possible, that as discussed above, the dancing together increased the awareness of the limitations in addition to the extra burden of the care-partner in organising the attendance of the classes (and the timely medication in preparation of these). Therefore, these points might have overshadowed some of the benefits that have arisen from attending the classes together.

Nevertheless, there is thus strong evidence that movement synchronicity is an important factor in a couples’ relationship. When the communication between couples is impaired, a connection through the moving body still allows the experience of many aspects of a relationship, such as the possibility of interactive playfulness. Overall, our data showed indeed that the participatory experiences in dance classes for Parkinson’s play a special role in the focus on the couples’ wellbeing. In line with previous studies that found including dance and movement in couple therapy is beneficial to couples’ experiences of intimacy, communication, closeness, and playfulness in general (e.g., Shuper Engelhard, 2018; Shuper Engelhard and Vulcan, 2018), we found supporting evidence that moving together is also experienced as beneficial for the psychological and emotional wellbeing for couples with care responsibilities in regards to playfulness, understanding of the disease, and a sense of care.

### Limitations and Future Recommendations

This study is not likely to be representative of the general population living with Parkinson’s disease, as the participants were recruited from affluent areas of Greater London with the privilege to attend private dance classes. Most people living with a progressive disorder in the United Kingdom do not have these extra funds available to them and might therefore have a very different experience of living with Parkinson’s. For future studies, we thus recommend using a more diverse sample. Ideally, dance for Parkinson’s programmes would be funded in order to allow free participation in more deprived areas as it is possible that people who cannot afford to pay for private classes would benefit just as much from a dance intervention, provided that they would be interested in this kind of class. Local experience from Scotland showed that free Parkinson’s dance classes can attract a mixed socio-economic group. For this study, it was noted that not all participants with Parkinson’s used to dance before, and that the stigma attached to dance was present but could be overlooked. I thus seem feasible that with playful interactive dance classes that use music choices which speak directly to the participants and instructors that are being understood by participants, can raise an interest in various groups. Yet whilst the impact of the current study is important and applicable to those living with Parkinson’s, the findings are not aiming to be representative of others’ experiences of living with Parkinson’s. Importantly, those from disadvantaged socioeconomic backgrounds may not have a care-partner or one that is supporting the dancing and they may have more comorbidities as a result of healthcare inequalities, in addition to other language and cultural barriers. Secondly, in the current sample, only one participant was middle aged, and the rest of the participants were of older age. There is a chance that aged-matched dance groups would be beneficial for younger individuals as they could potentially relate to one another more easily.

Another avenue for future research around dance for Parkinson’s is to study the impact dance classes have on empathy and emotion expression quantitatively. We highlighted the importance of empathic abilities in romantic relationships and the challenges Parkinson’s causes in that regard but they did not suffice as themes. Whilst one Parkinson’s participant showed their enjoyment of expressing familiar gestures, no specific experiences were expressed associated with changes in empathy through the dancing. A recent review by Pick et al. (2019) showed that research regarding the understanding of empathic deficits in Parkinson’s is sparse and fragmented, despite its importance in the establishment of a functional doctor-patient relationship. With reference to the care-partners wellbeing, it is of interest to note people who engage in embodied and theoretical practices, such as theatre, show higher empathic skills without increased feelings of distress (e.g., Schmidt et al., 2021). Dance classes that emphasise a theatrical component could therefore be beneficial not only for people with Parkinson’s through the practice of gestures and facial expressions but also for their care-partner’s resilience. We know that people with Parkinson’s benefit from the presence of empathic carers (Pomponi et al., 2016). We suggest that it would make a huge difference for care-partners’ sense burden were they able to share emotions in response to negative events without letting it overly affecting their own feelings. Yet more research is needed to address these potentials of dance classes for people with Parkinson’s and their care-partners.

Finally, some participants also had severe communication difficulties and might have had more to say than they were physically able to on the day of interviewing. Some of the interview questions were also too long and complicated for the people with Parkinson’s to process. Furthermore, the care-partners were asked mainly about their experiences with Parkinson’s and their relationship with their partner rather than about themselves as a person and carer. This could have led to limited findings as the care-partners may have been primed to only focus on Parkinson’s during the interview and therefore refrained from talking about their personal concerns or non-Parkinson’s parts of their lives.

## Conclusion

Parkinson’s participants experiences were found to be related to the questioning of experienced changes with the disease as well as the dancing. The importance of knowledge in general as well as in regard to the instructors’ expertise was evident. Other themes were the need for seeing and being seen to feel validated and included, as well as the ability of dance to act as a social enabler and mood changer. Further, albeit limited, we found evidence that dance classes provide motor skills training that is transferrable to everyday tasks. We did not expect the consistent prevalence of humour people with Parkinson’s showed throughout the interviews nor did we anticipate the importance this group placed on the playfulness interaction in dance classes as well as with their family or friends. The group aspect was particularly important to the care-partners as they were able to interact with others who have Parkinson’s and gain an insight into how their life may change in the future when their partner deteriorates further. And whilst the current sample was small, the dance classes were an avenue through which the care-partners were able to have an enjoyable moment as a couple and create happy memories together despite the looming prognosis of Parkinson’s. We deliberately did not ask our participants directly about the impact of dance on Parkinson’s or their relationship to give them more freedom to discuss their personal lived experiences and thus reduce the repetition of preconceived ideas. It is possible that our approach reduced the insight into specific aspects of dance for Parkinson’s but considering the vast number of studies on the benefits of dance for Parkinson’s we feel that a wider more participant-led perspective provided us with a better understanding of the conditions surrounding the role dance plays in their lives.

## Reflective Statement

Both authors have practical experience with dance for Parkinson’s. The first author also has professional experience in care for the elderly and people with dementia. Nevertheless, they found some of the interviews challenging due to the lack of facial expressiveness in some interview partners, which is often present in people with Parkinson’s. Due to this, the interviewer found it difficult to judge whether a participant was upset or not in response to some of the questions and when required diverted to asking about their experiences of Parkinson’s in general to try and prevent participant distress. This approach was chosen based on the researcher’s previous work experience and extensive training in how to talk to and interview people with cognitive impairments such as those seen in people with Parkinson’s. The second author is a scientist with professional experience in dance. Despite or because of them loving the art of dance, they pay particular attention to not only highlight the benefits of dance but also look at aspects of dance that might be lacking or problematic. The questions were thus designed in a way that allowed participants as much as possible to discuss dance from their personal experience in an authentic, truthful, and wholesome manner. The second author was not present at the interviews but participated in the analysis based on the transcripts.

## Data Availability Statement

There are some limits to our ability to share raw data in full, as identified by our local ethics committee as a condition of ethical approval. However, we are able to consider requests to share fully anonymized extracts of the data relevant to the study.

## Ethics Statement

The studies involving human participants were reviewed and approved by School of Applied Sciences Research Ethics Committee, Abertay University, Reference EMS1835. The patients/participants provided their written informed consent to participate in this study.

## Author Contributions

MS generated the idea of the study, conducted the interviews, was responsible for the first thematic analysis, and wrote the main parts of the first manuscript version. CJ oversaw the running of the study, advised on all methodological issues, in particular participant access and the interview schedule, and was responsible for the revision of the manuscript. Both authors conducted the second analysis together.

## Conflict of Interest

The authors declare that the research was conducted in the absence of any commercial or financial relationships that could be construed as a potential conflict of interest.

## Publisher’s Note

All claims expressed in this article are solely those of the authors and do not necessarily represent those of their affiliated organizations, or those of the publisher, the editors and the reviewers. Any product that may be evaluated in this article, or claim that may be made by its manufacturer, is not guaranteed or endorsed by the publisher.
